# Endogenous advanced glycation end products in the pathogenesis of chronic diabetic complications

**DOI:** 10.3389/fmolb.2022.1002710

**Published:** 2022-09-15

**Authors:** Misganaw Asmamaw Mengstie, Endeshaw Chekol Abebe, Awgichew Behaile Teklemariam, Anemut Tilahun Mulu, Melaku Mekonnen Agidew, Muluken Teshome Azezew, Edgeit Abebe Zewde, Assefa Agegnehu Teshome

**Affiliations:** ^1^ Department of Biochemistry, College of Medicine and Health Sciences, Debre Tabor University, Debre Tabor, Ethiopia; ^2^ Department of Physiology, College of Medicine and Health Sciences, Debre Tabor University, Debre Tabor, Ethiopia; ^3^ Department of Anatomy, College of Medicine and Health Sciences, Debre Tabor University, Debre Tabor, Ethiopia

**Keywords:** glycation, advanced glycation end products, diabetes complication, hyperglycemia, receptor advanced glycation end products

## Abstract

Diabetes is a common metabolic illness characterized by hyperglycemia and is linked to long-term vascular problems that can impair the kidney, eyes, nerves, and blood vessels. By increasing protein glycation and gradually accumulating advanced glycation end products in the tissues, hyperglycemia plays a significant role in the pathogenesis of diabetic complications. Advanced glycation end products are heterogeneous molecules generated from non-enzymatic interactions of sugars with proteins, lipids, or nucleic acids *via* the glycation process. Protein glycation and the buildup of advanced glycation end products are important in the etiology of diabetes sequelae such as retinopathy, nephropathy, neuropathy, and atherosclerosis. Their contribution to diabetes complications occurs via a receptor-mediated signaling cascade or direct extracellular matrix destruction. According to recent research, the interaction of advanced glycation end products with their transmembrane receptor results in intracellular signaling, gene expression, the release of pro-inflammatory molecules, and the production of free radicals, all of which contribute to the pathology of diabetes complications. The primary aim of this paper was to discuss the chemical reactions and formation of advanced glycation end products, the interaction of advanced glycation end products with their receptor and downstream signaling cascade, and molecular mechanisms triggered by advanced glycation end products in the pathogenesis of both micro and macrovascular complications of diabetes mellitus.

## Introduction

Diabetes mellitus (DM) is a chronic metabolic condition marked by hyperglycemia caused by abnormalities in insulin production, action, or both (Punthakee et al., 2018). It is associated with both acute and long-term vascular complications affecting the eye, kidney, nerves, and blood vessels which are the major causes of mortality and morbidity (Forbes and Cooper, 2013). Exposure to a hyperglycemic environment is the underlying cause of the pathogenesis of diabetic complications by activating or raising the rate of several metabolic pathways such as; the polyol pathway, the Protein Kinase C (PKC) pathway, and the production of advanced glycation end products (AGEs) (Park et al., 2019).

AGEs are heterogeneous substances formed by irreversible non-enzymatic interactions between reducing sugars and proteins, lipids, or nucleic acids, a process known as glycation (Perrone et al., 2020). AGEs can be exogenous (derived from food) or synthesized in the body endogenously (Vadakedath and Kandi, 2018). Exogenous AGEs can be found in meals (especially in western diets) as a result of cooking or food processing. Cooking circumstances (high temperatures for an extended period of time, low hydration, and high pH) generate huge numbers of several classes of AGEs (Mastrocola et al., 2020). Endogenous AGEs are majorly produced via the complex Maillard reaction, in which aldehyde groups on reducing sugars such as glucose, fructose, and ribose undergo a series of non-enzymatic reactions to the terminal amino groups of proteins, nucleic acids, and phospholipids, resulting in the formation of reactive carbonyl compounds (Fishman et al., 2018).

Glycation is a spontaneous and slow process under physiological conditions because it does not involve an enzyme catalyst; it requires several days or weeks to get completed (Rabbani and Ahn, 2019). AGEs, on the other hand, are actively generated and accumulate in the circulating blood and numerous tissues in the case of chronic hyperglycemia (Ahmad et al., 2014; Vadakedath and Kandi, 2018). Recently, both exogenous and endogenous AGEs have been implicated in the development of diabetic vascular problems in several investigations (Uribarri et al., 2015; Rhee and Kim, 2018). One of the potential mechanisms linking inflammation-related diabetes consequences such as atherosclerosis is exogenous AGE-induced extracellular matrix (ECM) alteration (Garay-Sevilla et al., 2021). Aside from atherosclerosis, AGE accumulation in ECM is thought to be important in other DM complications, as it has been linked to an increased risk of retinopathy and renal failure (Koska et al., 2018). However, the scope of this paper is on endogenous AGEs and their contribution to diabetic complications.

Clinical studies have revealed a link between the accumulation of endogenous AGEs and the occurrence of vascular problems in diabetes individuals (Koska et al., 2017). As indicated in Table 1, a significant association between AGEs and diabetic complications has been demonstrated. Furthermore, the measurement of AGEs in the tissue or circulation might be considered a promising biomarker and suggested as a predictor of diabetic complications (Simó-Servat et al., 2018). However, the precise biochemical processes underlying the pathophysiology of AGE-induced diabetic complications need to be elucidated. Hence, the focus of this paper was to compressively discuss; the chemical reactions and formation of AGEs, the interaction between AGE with their receptor and downstream signaling cascade, and molecular pathways induced by AGEs in the pathogenesis of both micro and macro-vascular complications of DM.

**TABLE 1:** Clinical and observational studies demonstrating the association between tissue or circulating AGEs and/or RAGE with diabetic chronic complications.

Author (s)	Year	Country/setting	Study subjects	Study design	Sample/measurement	Major findings
Barriquand, Romain, et al. (Barriquand et al., 2022)	2022	France	196 T1 DM patients	Cross-sectional study	Tissue/skin AF	Increased circulation/tissue AGEs were associated with both micro and macrovascular complications in DM patients
Farhan, Sinan, et al. (Farhan and Hussain, 2019)	2019	Jordan	50 T2 DM patients	Comparative cross-sectional study	Serum/ELISA	DM patients with complications had significantly higher serum levels of AGEs and AGEs/RAGE ratio than patients without complications and healthy controls. AGEs can be an early predictor of Reno-vascular complication
Takayanagi, Yuji, et al. (Takayanagi et al., 2020)	2020	Japan	229 DM patients and 165 healthy controls	Comparative cross-sectional study	Tissue/skin AF	AGEs were independently associated with the progression of diabetic retinopathy
Kopytek, Magdalena, et al. (Kopytek et al., 2020)	2020	Poland	126 T2 DM patients	Prospective observational study	Serum/ELISA	Accumulation of AGEs in DM patients is associated with the severity of aortic stenosis
Thomas, Merlin, et al. (Thomas et al., 2015)	2015	A multi-centered study	3,763 T2 DM patients	Case-cohort study	Serum/ELISA	Increased levels of AGEs and soluble RAGE are independently associated with new onset or worsening of nephropathy in DM patients
Hangai, Mari, et al. (Hangai et al., 2016)	2016	Japan	122 T2 DM patients	Cross-sectional study	Tissue/skin AF	Accumulation of AGEs was positively correlated with coronary artery calcification
Chawla, Diwesh, et al. (Chawla et al., 2014)	2014	India	75 T2 DM patients	Cross-sectional study	Serum/ELISA and PCR	AGEs level and RAGE mRNA expression were significantly higher in patients with vascular complications than without complication
Rigalleau, V, et al. (Rigalleau et al., 2015)	2015	France	418 T2 DM patients	Cross-sectional study	Tissue/skin AF	Accumulation of AGEs was independently associated with chronic kidney disease and macroangiopathy
Paul J et al. (Beisswenger et al., 2013)	2013	USA	103 T1 DM patients	Cross-sectional study	Plasm/Liquid chromatography	Diabetes nephropathy patients had significantly higher levels of AGEs. AGEs may also be early indications of diabetic nephropathy
Ying, Lingwen et al. (Ying et al., 2021b)	2021	China	1006 T2 DM patients	Cross-sectional study	Skin/AF	AGEs via skin AF is a potential marker of carotid atherosclerosis in T2 DM patients

Abbreviations: AF, Auto-fluorescence; ELISA, enzyme linked immunosorbent assay; PCR, polymerase chain reaction.

## Biochemistry of AGEs

### Biosynthesis of AGEs

The synthesis of AGEs is a complicated molecular process involving a multistep reaction. The whole process in the formation of AGE is called the Maillard reaction (Popova et al., 2010). Louis-Camille Maillard (1878–1936) developed the process in 1912, reporting that mixes of amino acids and sugars become intensely brown at high temperatures. After 40 years, chemist John E. Hodge discovered the mechanism of the Maillard reaction (Hellwig and Henle, 2014). It is a series of cascade reactions that occurs between free amino groups of proteins, peptides, and amino acids with carbonyl groups of reducing sugars (Perrone et al., 2020). This reaction has three distinct stages: early, intermediate, and late. A reducing sugar, such as glucose, combines non-enzymatically with the free amino group of protein to create the Schiff base in the early stages of the Maillard process. The Schiff base is then subjected to a rearrangement process, yielding a more stable molecule known as the Amadori product. In an intermediary stage, dehydration, oxidation, and other chemical events progressively degrade the Amadori product (keto-amine) to a variety of reactive carbonyl and dicarbonyl molecules such as glyoxal, methylglyoxal (MGO), and deoxyglucosones (Zhang et al., 2010; Tsekovska et al., 2016; Kim et al., 2017). Dicarbonyl compounds are the primary precursors for the synthesis of several key flavor products, heterocyclic compounds, and polymers. The highly electrophilic nature of these dicarbonyl compounds makes them react relatively faster with guanidine, arginine, lysine, and sulfhydryl functional moieties of proteins to produce different irreversible adducts (Allaman et al., 2015). Through polymerization, oxidation, dehydration, and cyclization events, nearly irreversible compounds known as AGEs are generated in the late or final stage of the Maillard reaction (Tsekovska et al., 2016). The properties of the participating reactants are one of the factors that affect the Maillard reaction. For instance, the monosaccharide fructose is more reactive *in vitro* than glucose (Yu et al., 2018). AGE molecules have the capacity to interact with certain proteins, leading to cross-links that impair the function of the body’s cells and tissues (Indyk et al., 2021).

Although the Maillard reaction is the most common process for the synthesis of AGEs, there are also other minor pathways, such as the polyol pathway and lipid peroxidation (Kuzan, 2021). The polyol pathway contains two enzymatic reactions catalyzed by aldose reductase (AR) and sorbitol dehydrogenase. Aldose reductase, the rate-limiting enzyme, reduces glucose to sorbitol at the expense of reduced nicotinamide adenine dinucleotide phosphate (NADPH), while, sorbitol dehydrogenase converts sorbitol to fructose at the expense of NAD^+^ leading to reduced nicotinamide adenine dinucleotide (NADH) production (Tang et al., 2012; Mathebula, 2015). Then the accumulated fructose can then be transformed into 3-deoxyglucose and fructose-3-phosphate, both of which are extremely powerful non-enzymatic glycation agents (Gugliucci, 2017). However, not only the polyol pathway (endogenous fructose), but consumption of fructose-rich diets (exogenous fructose) can also result in the formation of fructose-mediated AGEs (Sotokawauchi et al., 2019). Another process implicated in the synthesis of endogenous AGEs and protein cross-linking is lipid peroxidation. Lipid peroxidation is a process in which oxidants such as free radicals deteriorate lipids that contain a carbon-carbon double bond (Ayala et al., 2014). Oxidative stress-induced increased production of reactive aldehydes can occur because of lipid peroxidation and glycoxidation. Consequently, protein cross-linking, oligomerization, and the production of protein oxidation adducts occur (Moldogazieva et al., 2019).

The normal physiological rate of AGEs synthesis is proportional to the rate of protein turnover and oxidative stress. Long-lived proteins (such as collagen and elastin) are more vulnerable to glycation (Guo and Xu, 2017). The extent of AGEs synthesis *in vivo* is also increased by substrate availability (i.e., monosaccharides). As a result, their rate of production is accelerated in hyperglycemic conditions (Khalid et al., 2022). Although there are, several AGEs compounds that have not been well characterized, carboxymethyl-lysine (CML) and pentosidine have been the most described and studied AGEs so far (Ng et al., 2013a; Ashraf et al., 2014; Ashraf et al., 2015). They are insoluble, yellow-brown in appearance, and have fluorescent features that allow them to be detected in circulation and tissues. In general, AGEs can be classified as fluorescent or non-fluorescent based on their features (Haddad et al., 2016).

### AGE and its receptor (RAGE) interaction

AGE receptors are known as receptor advanced glycation end-products (RAGEs), which are signal transduction receptors. It is a multi-ligand trans-membrane protein belonging to an immunoglobulin superfamily that is encoded by a gene on chromosome 6 and has 394 amino acids (Chawla and Kumar Tripathi, 2019). RAGE gene expression is abundant in many tissues and cells, including the vasculature, lung, heart, brain tissue, smooth muscle cells, monocytes/macrophages, and endothelial cells (Perrone et al., 2020). The full length of RAGE is the most well-studied isoform and has three domains; extracellular, transmembrane, and cytosolic domains (Chuah et al., 2013). Alternative splicing of mRNA of RAGE also leads to an additional RAGE isoform called soluble or secretary RAGE (sRAGE) which is a freely circulating form lacking a trans-membrane domain and not involved in the pathogenesis effect of AGEs (Ng et al., 2013b). Rather, they bind to AGEs to ensure endocytosis and degradation for the removal of AGEs (Kay et al., 2016).

**FIGURE 1:**
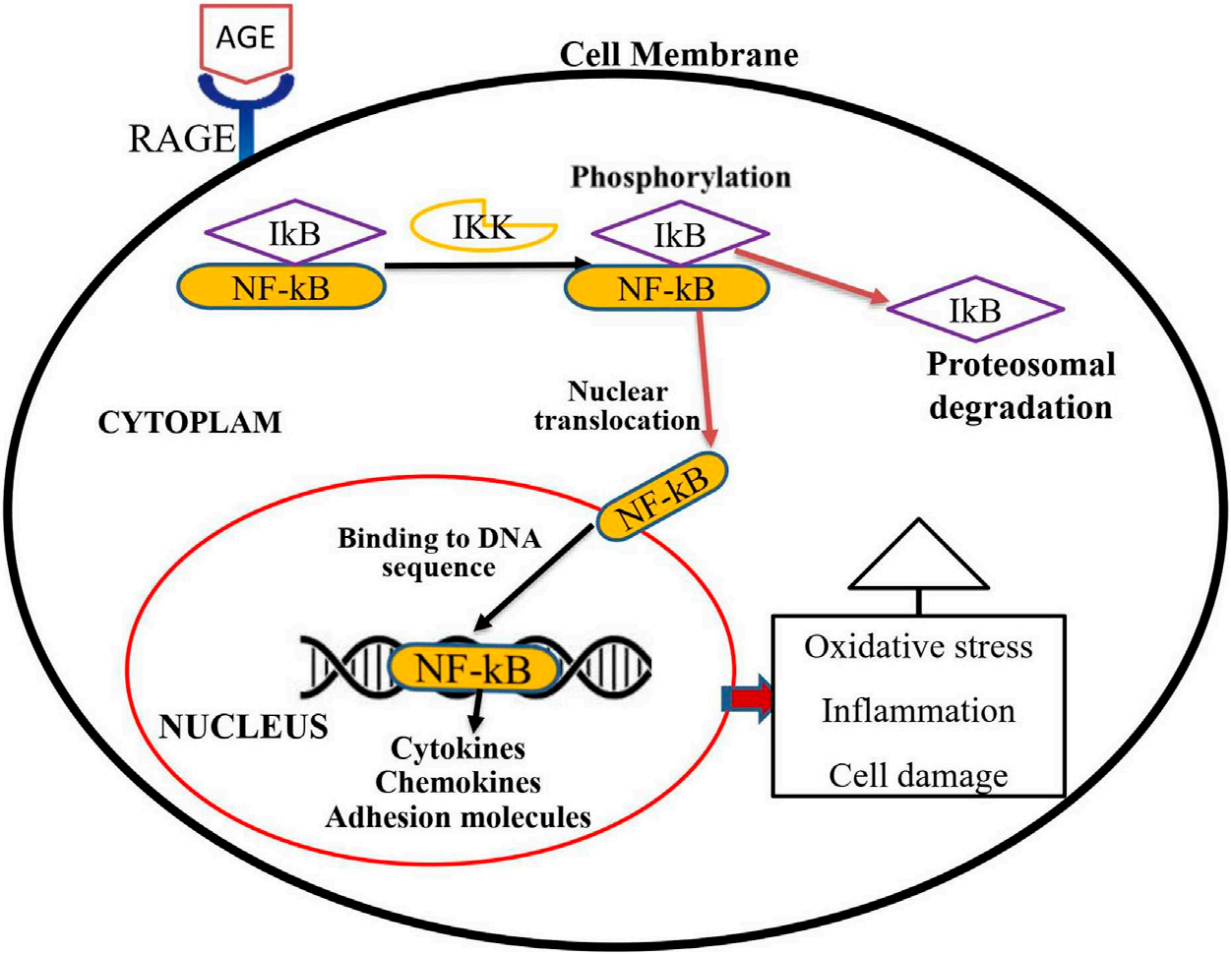
Activation process of NF-κB transcription factor through an AGE-RAGE signaling pathway. Activation of RAGE through AGE interaction transduces a signal for the phosphorylation of IkB by IKK. Phosphorylated IkB then could be detached from the cytosolic NF-kB transcription factor. Multiple genes such as cytokines, chemokines, and adhesion molecules are activated when active and free NF-kB translocate into the nucleus. These proteins trigger oxidative stress, inflammation, and cellular damage, all of which contribute to diabetic complications.

The interaction of AGE and RAGE is critical in the pathophysiology of many diseases, including diabetes complications. The activation of RAGEs by AGEs transduces various signals, including those from the mitogen-activated protein kinases (MAPKs), extracellular signal-regulated kinases (ERKs), and Janus kinase, resulting in inflammatory, angiogenic, proliferative, and apoptotic responses (Chawla and Kumar Tripathi, 2019). The most frequently researched AGE receptor isoform is full-length RAGE, which is abundantly found on vascular endothelial cells. Through the activation of nuclear factor (NF-κB), the recognition of AGE by RAGE in endothelial cells accelerates the production of oxidative stress, different cytokines, and growth factors, culminating in the generation of inflammation (Rhee and Kim, 2018). The AGE/RAGE signaling cascade also increases the formation of reactive oxygen species (ROS) by activating certain signaling pathways such as TGF-α and NADPH-oxidase 1 (Nox-1). ROS-induced oxidative stress can alter a variety of intracellular components, including cellular membrane proteins, lipids, and DNA (Bongarzone et al., 2017). NF-κB is a universal transcription factor normally found in an inactive form in the cytoplasm bound to its inhibitor protein called IκB (Sivandzade et al., 2019). As illustrated in Figure 1, phosphorylation of IκB by a kinase protein called IκB kinas (Ikk), results in the separation of IκB from NF-κB. Then NF-κB trans-locates to the nucleus and binds to a specific region of DNA, triggering the development of numerous cytokines, chemokines, cell adhesion molecules, interleukins, TGF, pro-inflammatory proteins, and pro-apoptotic genes (Huang and Hung, 2013; Hinz and Scheidereit, 2014; Suryavanshi and Kulkarni, 2017).

### AGEs in the pathogenesis of diabetic complication

**FIGURE 2:**
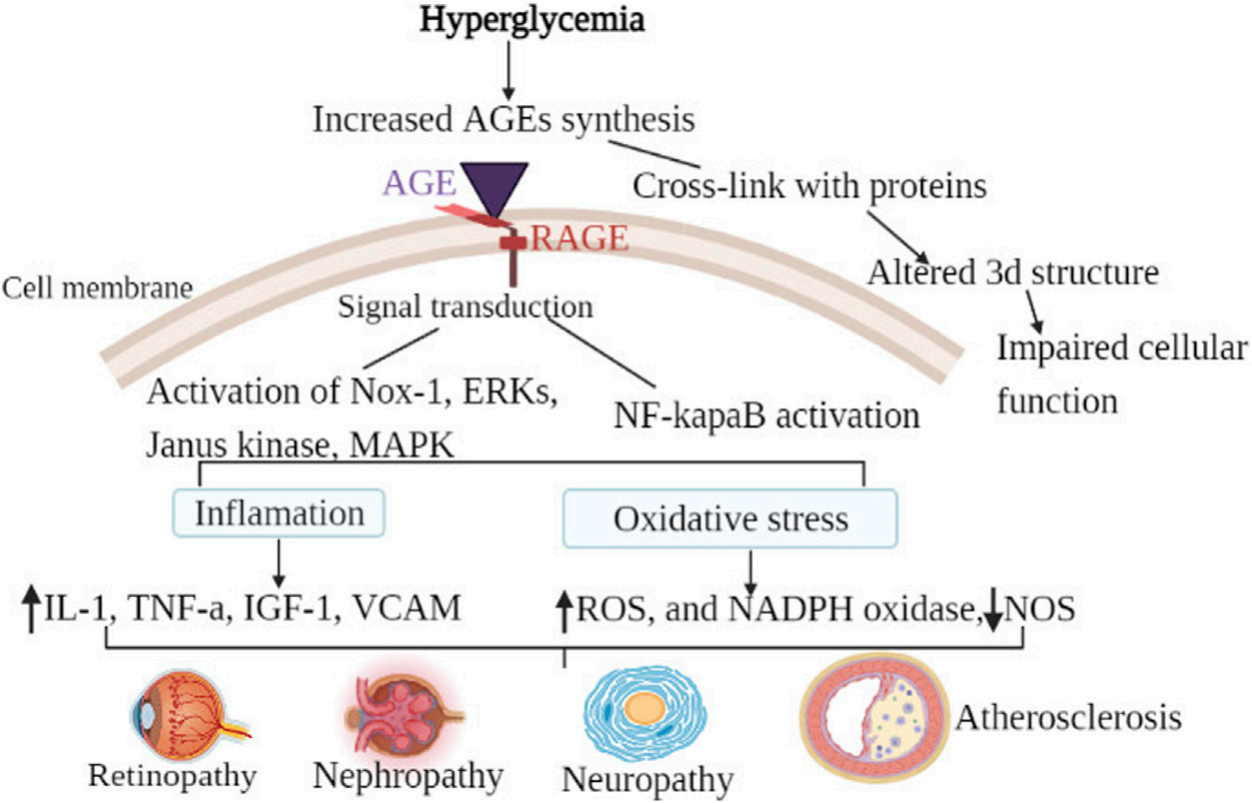
General mechanism of AGEs in diabetic chronic complications. Hyperglycemia is the most common cause of the synthesis of endogenous advanced glycation products in diabetic patients. The general mechanism of advanced glycation end-products in diabetic vascular complications is due to the activation of multiple signal transduction pathways as a result of RAGE/AGE interaction or through cross-link formation with cellular proteins. Activation of RAGE leads to the activation of Nox-1, ERKs, Janus kinase, MAPK, and activation of NF-κB. When those pathways are activated, oxidative stress (decreased NOS, increased ROS, and increased NADPH oxidase) and inflammatory factors are activated. Cross-link (adduct) formation on the other hand altered the three-dimensional structure of protein consequently impairing cellular function. As a result of the combined effect, diabetic vascular complications such as retinopathy, neuropathy, nephropathy, and atherosclerosis develop.

The chief causes of mortality and morbidity in diabetes are vascular complications, particularly microvascular and cardiovascular complications (Barrett et al., 2017). As previously stated, AGE cross-links are permanent and irreversible complexes generated when glucose binds to target proteins. As a result, once generated, AGEs will remain and continue to harm the tissue until the proteins involved are destroyed (Guo and Xu, 2017). AGEs can interact with the RAGE receptor to cause a variety of adverse outcomes including oxidative stress, apoptosis, and inflammation, as well as build the so-called “hyperglycemia memory” (Khalid et al., 2022). Although additional mechanisms, such as oxidative stress and epigenetic modifications, are implicated in the pathophysiology of hyperglycemic memory (currently metabolic memory), AGEs are the key contributors to metabolic memory (Zhan et al., 2022). Glycation and oxidative stress are inextricably related processes that are frequently referred to as glycoxidation. AGEs enhance the generation of reactive oxygen species (ROS) and impede antioxidant mechanisms; nonetheless, certain AGEs are formed naturally under oxidative conditions (Nowotny et al., 2015). Furthermore, AGE appears to be the primary driver of microvascular problems in DM for at least two reasons: First, AGE has various intra- and extracellular targets. Second, regardless of hyperglycemia level, Age-related intracellular glycation of mitochondrial respiratory chain proteins has been shown to produce additional reactive oxygen species, creating a vicious cycle that promotes AGE synthesis (Chilelli et al., 2013). As shown in the Figure 2 the general mechanisms by which AGEs contribute to all types of diabetic complications are either; receptor-mediated signaling cascade (AGE/RAGE cell surface interaction) or damage to the extracellular matrix through their cross-linking nature (Singh et al., 2014). Hence, understanding the mechanisms underlying accelerated diabetic complications is essential to uncover so that targeted therapeutic strategies might be developed.

### AGEs in diabetic retinopathy

Diabetic retinopathy (DR) is the most prevalent cause of blindness in people with DM, and it is characterized by lesions within the retina caused by changes in vascular permeability, capillary micro-aneurysm, loss of pericytes, and excessive development of new blood vessels (angiogenesis) (Shin et al., 2014). Recent epidemiological investigations have demonstrated that AGEs are linked to both the prevalence and severity of DR in diabetic patients (Beisswenger et al., 2013; Ying et al., 2021a). A significant association between skin AGEs and DR staging in T2DM patients was also reported, and skin AGE levels could substantially predict the degree of DR (Zhang et al., 2022). Chronic hyperglycemia exposure causes AGE buildup in the retina (Alan, 2018). Accumulation of AGEs in the retinal endothelial microcirculation contributes to premature closure (occlusion) of capillaries (Xu et al., 2018). Furthermore, they generate an increase in intracellular cell adhesion molecules (ICAM), which mediates retinal capillary leukocyte adherence and the collapse of the inner blood-retinal barrier, culminating in retinal injury (Moore et al., 2003; Alan, 2018).

Increasing pieces of evidence suggested that mRNA of RAGE is highly expressed in retinal cells of diabetic patients due to increased AGEs (Rujman Khan, 2016). Circulating AGE levels are positively linked with RAGE mRNA expression and oxidative indicators in patients with type 2 DM (Chawla et al., 2014). This means that RAGE expression could be enhanced in settings where ligands and inflammatory mediators accumulate (Yaw et al., 2013). It has been demonstrated that in a ligand-enriched environment (AGE accumulation), RAGE expression may rise and proinflammatory processes may be exacerbated (Senatus and Schmidt, 2017). The binding of AGE to RAGE triggers critical signaling pathways such as tyrosine phosphorylation of Janus kinase (JAK)/signal transducers and activators of transcription (STAT), activation of protein kinase C, and oxidative stress *via* NF-κB (Ott et al., 2014). Finally, it increases the expression of adhesion molecules and the production of cytokines including tumor necrosis factor-alpha (TNF-α) and vascular endothelial growth factor (VEGF) (Safi et al., 2014). Cytokines such as IL-α, IL-β, and IL-6 are mediators of inflammation in the retina whereas; VEGF is involved in the formation of new blood vessels (angiogenesis) in the retinal endothelium, which contributes to proliferative retinopathy (Gui et al., 2020). AGEs, on the other hand, independently promote the secretion of IL-6 from retinal cells, which can lead to neovascularization by boosting VEGF expression (Alan, 2018). It was also demonstrated that AGEs could enhance mRNA expression of TNF-α but reduce eNOS mRNA expression in human endothelial cells, which may contribute to vascular dysfunction in DM (Rashid et al., 2004).

Recently, RAGE gene variants (gene polymorphism) have been associated with DR by altering RAGE gene expression, albeit there are some contradictory studies (Tao et al., 2017). For instance, 2245 G/A RAGE gene polymorphisms were associated with the development of DR in the Malaysian population (Ng et al., 2012a). 374 T/A RAGE gene polymorphism might be also a risk factor for DR among Pakistani T2 DM patients (Qayyum et al., 2021). On the other hand, another study conducted on Malaysian DM patients reported that -429 T/C and -374 T/A gene polymorphism in the promoter region of the RAGE gene were not associated with DR (Ng et al., 2012b). There was also no association between 1704 G/T and 2184 A/G RAGE gene polymorphism and retinopathy susceptibility in DM patients (Ng et al., 2012c). Hence, more research is required to determine the relationship between RAGE gene polymorphism and DR.

AGEs also play an important part in the loss of lens transparency (cataract formation) (Hashim and Zarina, 2017). A cataract is one of the most common causes of vision impairment in diabetics. Glycation of lens proteins (crystallins) has been identified as one of the mechanisms causing diabetes cataracts. AGEs cause permanent alterations in structural proteins, causing lens protein aggregation and the creation of high-molecular-weight aggregates, which causes light dispersion and impairs vision (Rujman Khan, 2016). *In vitro* investigations also revealed that AGEs in pericytes promote apoptosis by increasing the activity of caspase-3 due to a reduction in the Bcl/Bax ratio (Safi et al., 2014). Because pericytes play a vital role in the maintenance of microvascular homeostasis, their loss can cause endothelial cell damage and angiogenesis in the retinal blood vessels, resulting in diabetic retinopathy (Huang, 2020).

### AGEs in diabetic nephropathy

Diabetic nephropathy (DN) is the leading cause of end-stage renal failure in diabetic people (Lin et al., 2018). Clinically, it is defined by the development of proteinuria, followed by a gradual reduction in the glomerular filtration rate over time. It is also a substantial risk factor for macrovascular problems if left untreated (Parwani, 2017; Lin et al., 2018). AGE levels in renal tissue have been found to correlate with DN. AGEs disrupt the balance between synthesis and degradation of glomerular basement membrane extracellular matrix (ECM) components, particularly collagen (Anil Kumar et al., 2014). The cross-linking of AGE with collagen in the basement membrane will contribute to membrane thickening, impaired filtration, and eventually loss of glomerular function (Pasupulati et al., 2016).

AGE-RAGE axis also plays an important role in DN. TGF-β expression is stimulated by AGE-RAGE signaling in podocytes, tubular cells, and mesangial cells (Ott et al., 2014). TGF-β is expressed via the JAK/STATA signaling pathway and is a pro-fibrotic factor that increases the production of type IV collagen, laminin, and fibronectin, causing basement membrane thickening (Singh et al., 2014). The other mechanism of DN is through cross-talk between AGEs and Rennin-Angiotensin-Aldosterone System (RAAS). RAS components include renin, angiotensin I, angiotensin-converting enzyme (ACE), and angiotensin II, which is predominantly recognized to regulate fluid balance (Lin et al., 2018). Angiotensin II causes mesangial and tubular epithelial cell hypertrophy and acts via the angiotensin II type 1 receptor (AT1R). AGEs, on the other hand, increase AT1R expression, which increases Angiotensin II activity (Parwani, 2017).

Furthermore, AGE also induces renal inflammation and fibrosis. Activation of RAGE induces the expression of various cytokines in kidney cells as well. These cytokines, in turn, promote monocyte chemoattractant protein-1 (MCP-1) production in renal cells, which is related to monocyte/macrophage infiltration into the cell (Nowotny et al., 2015). In addition, AGEs also induce podocytopathy. Glomerular podocytes are specialized cells that act as a size-selective filtration barrier, regulating the entry of plasma proteins into the urine from circulation. RAGE activation in podocytes promotes NF-κB signaling, which causes zinc finger protein production called homeobox-2 E-box binding (ZEB2) (Pasupulati et al., 2016). ZEB2 is a transcription factor that regulates the epithelial-mesenchymal transition by inhibiting E-cadherin (an epithelial marker) and activating N-cadherin (a mesenchymal marker). Podocytes may undergo epithelial-mesenchymal transition and detach from the basement membrane, resulting in a drop in podocyte count per glomerulus and proteinuria (Loeffler and Wolf, 2015). A significant association between AGEs, particularly CML, with podocyte injury and proteinuria was also observed in DM patients, which contributed to impaired kidney function (Nishad et al., 2021). However, the precise signaling mechanisms of NF-κB/ZEB2 in podocytes still need to be explored.

### AGEs in diabetic neuropathy

Diabetic neuropathy is a phenomenon characterized by segmental demyelination and axonal degradation in both the somatic and autonomic divisions of the peripheral nervous system (Salahuddin et al., 2014). It causes functional abnormalities such as decreased nerve transmission and blood flow, which increases the risk of lower extremity amputations in diabetics throughout the course of their lives (Amin and Doupis, 2016). The generation of AGEs in peripheral nerves has recently been identified as an additional risk factor for diabetic neuropathy development. The glycation of myelin is increased in diabetes. Glycated myelin is vulnerable to macrophage phagocytosis and induces macrophages to release proteases, which may contribute to nerve demyelination (Khangholi et al., 2015). It has been also observed that AGE modification of important axonal cytoskeletal proteins such as tubulin, neurofilament, and actin results in axonal atrophy/degeneration and decreased axonal transport (Sugimoto et al., 2008). Furthermore, *in vitro* studies revealed that oxidative stress increases the glycation of the Na+/K+ ATPase protein. Glycation of Na+/K+ ATPase may cause it to lose activity, resulting in a decrease in motor nerve conduction velocity (Singh et al., 2014). AGEs are elevated in the presence of distal sensorimotor polyneuropathy (DSPN) among T2 DM patients, and AGEs are linked with the severity of DSPN, according to observational studies (Papachristou et al., 2021). Decreased relative muscle strength was also observed in patients with T2 DM with elevated serum levels of AGEs (Wu et al., 2022).

### AGEs in diabetic atherosclerosis

Atherosclerosis is the most serious long-term diabetes complication, defined by the deposition of atherosclerotic plaque on the interior of artery walls, leading to blockage of blood flow and, finally, myocardial infarction/cardiomyopathy (Nowotny et al., 2015). Increased glycation of Apolipoprotein-B and phospholipid components of low-density lipoprotein (LDL) particle occurs in diabetes. Apo B, a surface protein of LDL, is glycated at a positively charged lysine residue in the receptor-binding domain. Glycated LDL is thus not recognized by the LDL receptor, yet its absorption by macrophages is increased. This could hasten the production of foam cells seen in diabetics (Knott et al., 2019). Glycation, on the other hand, increases the turnover of high-density lipoprotein (HDL) and lowers its efficiency during reverse cholesterol transfer. Furthermore, glycation of HDL decreases the activity of paraoxonase, an HDL-associated enzyme that prevents LDL oxidation and monocyte adherence to endothelial cells. Both of which are critical early stages in the production of atherosclerotic plaques (Komplikacija and Tipa, 2015).

It has been reported that AGE-RAGE signaling generates oxidative stress and inhibits endothelial cell-derived nitric oxide (NO). Endothelial dysfunction, an early hallmark of atherosclerosis, could be exacerbated by impaired endothelial cell-derived NO production and/or bioavailability (Yamagishi and Matsui, 2018). The cross-linking of AGEs with extracellular matrix proteins like collagen and elastin has also been linked to arterial stiffness. Through their interaction with RAGE, AGEs also increase the synthesis of vascular endothelial growth factor (VEGF) in endothelial cells. VEGF then promotes pathologic angiogenesis in the atheroma and exacerbates plaque inflammation (Rhee and Kim, 2018).

When AGE binds to receptors, it causes oxidative stress, nuclear factor (NF-κB) activation, and the synthesis of adhesive molecules and vascular cell adhesion molecules (VCAM-1), resulting in greater permeability of endothelial cells and a more intense invasion of lipids in the sub-endothelium (Milstone et al., 2016). AGEs in DM also activate the RAGE/toll-like receptor 4 (TLR4) pathway in plaque macrophages. Delta-like ligand 4 (DLL4) expression is promoted by activated RAGE/TLR4 signaling. Dll4 expression on macrophage-treated vascular smooth muscle cells (VSMCs) results in contractile-phenotypic conversion via the Notch pathway, which contributes to atherosclerosis (Xing et al., 2022). Additionally, AGE-RAGE interaction in monocytes-macrophages increases the synthesis of the following mediators: interleukin-1 (Il-1), tumor necrosis factor (TNF-a), platelet-derived growth factor (PDGF), and insulin-like growth factor-1 (IGF-1), all of which play a role in the pathogenesis of atherosclerosis (Komplikacija and Tipa, 2015). Furthermore, oxidative stress induced by AGE-RAGE signaling has been demonstrated to reduce the expression levels of Adenosine triphosphate Binding membrane Cassette transporter A1 (ABCA1) in cultured macrophages and eventually suppress cholesterol efflux from macrophages to Apo-A1 of High-Density Lipoprotein (HDL), ultimately impairing the reverse cholesterol transport system (Yamagishi and Matsui, 2018).

Given the detrimental effects of AGEs, ongoing studies are being carried out to develop molecules that can suppress the AGE-RAGE signaling pathway (Hong Sheng et al., 2017a). Drugs clinically approved for other indications such as statins, anti-hypertensive medicines, and anti-diabetic therapies are the most recent promising anti-AGEs-RAGE signaling agents (Nenna et al., 2015). Nevertheless, the clinical evidence on the anti-AGE-RAGE antagonizing action of these medications is still limited to certain drug classes (Jud and Sourij, 2019). Statins have been demonstrated in studies to increase sRAGE levels by triggering RAGE shedding. Hence they suppress pro-inflammatory disease-promoting ligand/RAGE pathways (Quade-Lyssy et al., 2013). Although its exact molecular mechanism is not clearly elucidated, atorvastatin has been shown to have a direct inhibitory effect on AGE-RAGE expression in the aorta of rats, indicating that it may protect against diabetes-related atherosclerosis (Xu et al., 2014). It was observed that in DR patients on antihyperglycemic and antihypertensive medications, both NF-κB p65 and circulating MCP-1 levels, which are pro-inflammatory markers, were significantly reduced (Ng et al., 2013c). It is hypothesized that inhibiting the AGE-RAGE axis may be more advantageous in the early stages of diabetes, delaying the advancement of related vascular complications (Hong Sheng et al., 2017b). Furthermore, in recent years, natural compounds rich in bioactive elements (phytochemicals) have been shown to interact with AGEs and their related mediators via multiple signaling cascades, thereby limiting and inhibiting the course of diabetes (Parveen et al., 2021). The results of ongoing and future clinical trials may aid in defining the best therapeutic target for AGEs in diabetes complications.

## Conclusion

Hyperglycemia promotes the development of AGEs in diabetic patients. Increased glycation and AGE accumulation in tissues and serum contribute significantly to the pathophysiology of diabetic vascular problems such as retinopathy, nephropathy, neuropathy, and atherosclerosis. Recent evidence focused on the molecular mechanism of AGE-RAGE axis cellular signaling in diabetic complications besides their impact on long-lived extracellular proteins through non-receptor-mediated mechanisms. Several chemical compounds are produced that appear to stimulate intracellular signal transduction pathways for the production of pro-inflammatory and pro-sclerotic cytokines, thereby increasing oxidative stress and gene expression and leading to the development and progression of diabetic vascular complications. In the future, these mechanisms and molecular pathways may be the source of new therapeutic targets to prevent vascular complications in diabetes mellitus patients.
